# Immunization with merozoite surface protein 2 fused to a *Plasmodium*-specific carrier protein elicits strain-specific and strain-transcending, opsonizing antibody

**DOI:** 10.1038/s41598-019-45440-4

**Published:** 2019-06-21

**Authors:** Jacqueline S. Eacret, Donna M. Gonzales, Raymond G. Franks, James M. Burns

**Affiliations:** Center for Molecular Parasitology, Department of Microbiology and Immunology, Drexel University College of Medicine, 2900 Queen Lane, Philadelphia, PA 19129 USA

**Keywords:** Malaria, Parasite host response

## Abstract

Vaccine trials and cohort studies in *Plasmodium falciparum* endemic areas indicate that naturally-acquired and vaccine-induced antibodies to merozoite surface protein 2 (MSP2) are associated with resistance to malaria. These data indicate that *Pf*MSP2 has significant potential as a component of a multi-antigen malaria vaccine. To overcome challenges encountered with subunit malaria vaccines, we established that the use of highly immunogenic r*Pf*MSP8 as a carrier protein for leading vaccine candidates r*Pf*MSP1_19_ and r*Pf*s25 facilitated antigen production, minimized antigenic competition and enhanced induction of functional antibodies. We applied this strategy to optimize a r*Pf*MSP2 (3D7)-based subunit vaccine by producing unfused r*Pf*MSP2 or chimeric r*Pf*MSP2/8 in *Escherichia coli*. r*Pf*MSP2 formed fibrils, which induced splenocyte proliferation in an antigen receptor-independent, TLR2-dependent manner. However, fusion to r*Pf*MSP8 prevented r*Pf*MSP2 amyloid-like fibril formation. Immunization of rabbits elicited high-titer anti-*Pf*MSP2 antibodies that recognized r*Pf*MSP2 of the 3D7 and FC27 alleles, as well as native *Pf*MSP2. Competition assays revealed a difference in the specificity of antibodies induced by the two r*Pf*MSP2-based vaccines, with evidence of epitope masking by r*Pf*MSP2-associated fibrils. Rabbit anti-*Pf*MSP2/8 was superior to r*Pf*MSP2-elicited antibody at opsonizing *P. falciparum* merozoites for phagocytosis. These data establish r*Pf*MSP8 as an effective carrier for a *Pf*MSP2-based subunit malaria vaccine.

## Introduction

Throughout the past several years, great progress has been made towards the goal of malaria elimination and eventual eradication with the widespread implementation of a number of control strategies^1^. The distribution and use of insecticide treated bednets, indoor spraying of residual insecticides, rapid diagnosis and treatment of mild malaria and intermittent preventative treatment of vulnerable populations have all contributed to the global reduction in malaria. However, data from the most recent World Malaria Report indicate that the preceding decline in clinical cases of malaria and malaria-related deaths is beginning to level off in most regions and is even showing signs of reversal in others. These data are concerning and are likely a result of increases in drug-resistant parasites and insecticide-resistant mosquitoes, along with the challenge of sustaining multiple control programs in endemic areas^1^. Considering the immunogenicity and efficacy data from a recent phase 3 clinical trial of RTS,S, the most advanced subunit malaria vaccine candidate, it seems unlikely that deployment of this vaccine will dramatically shift these trends^2–5^.

Natural immunity to malaria is acquired over time following repeated exposure to the many antigens of multiple strains of *Plasmodium falciparum* blood-stage parasites^6^. Protection has been associated with antibody responses primarily targeting polymorphic, parasite-encoded erythrocyte membrane proteins and multiple antigens of invasive merozoites^7–9^. A few of these antigens have emerged as potential vaccine candidates^10^. In general, success with merozoite antigen-based vaccines has been limited due to the structural complexity of these antigens leading to difficulties in production, antigen polymorphism and the allele-specificity of protective responses, low immunogenicity and ultimately insufficient vaccine efficacy^11,12^. Approaches to overcome these challenges have focused on the identification of new targets, novel vaccine delivery platforms and adjuvants, as well as modification of dose and schedule of vaccine administration^11,13,14^. As most subunit vaccine candidates have been tested as single antigen formulations, concurrent immunization with multiple target antigens and/or multiple alleles of those antigens affords significant potential to increase overall protective efficacy and durability of vaccine-induced responses.

We have previously demonstrated the utility of *P. falciparum* MSP8 as a carrier protein for *P. falciparum* vaccine candidates for overcoming some of the challenges with recombinant antigen production and/or immunogenicity of multi-component formulations^15–17^. Through genetic fusion of the blood-stage vaccine candidate, *Pf*MSP1_19_, to the N-terminus of the conserved, highly immunogenic *Pf*MSP8, we were able to produce high yields of chimeric r*Pf*MSP1/8 antigen with properly folded EGF-like domains. This chimeric antigen approach prevented antigenic competition and the vaccine elicited high-titer antibodies specific for conformation-dependent, protective epitopes of *Pf*MSP1_19_^16^. Most significantly, antibodies elicited by immunization of mice, rabbits and non-human primates demonstrated potent activity in the standard *in vitro* growth inhibition assay^16,18^.

The strategy to use *Pf*MSP8 as a universal carrier protein was further applied to a leading transmission-blocking candidate, *Pf*s25, a structurally complex protein notoriously difficult to produce and purify. As compared to r*Pf*s25 alone, we showed that fusion of *Pf*s25 to the *Pf*MSP8 carrier (r*Pf*s25/8) significantly increased yield and quality of r*Pf*s25, without requiring refolding procedures to achieve proper conformation of its four EGF-like domains. Immunization with chimeric r*Pf*s25/8 induced high titers of antibodies against conformational epitopes of *Pf*s25 that exhibited potent transmission-reducing activity as measured in the standard membrane feeding assay^17^. The success with chimeric r*Pf*MSP1/8 and r*Pf*s25/8 vaccines with respect to production, folding, immunogenicity and induction of functional antibodies, prompted us to evaluate *Pf*MSP8 as a fusion partner for another leading blood-stage vaccine candidate, *P. falciparum* MSP2.

*Pf*MSP2 is a~25 kDa GPI-anchored protein abundantly expressed on the merozoite surface that has a presumed role in erythrocyte invasion and is essential for parasite growth^19–23^. It is characterized by conserved N- and C-termini flanking highly polymorphic central variable regions (CVR), which can be classified into two major allelic families (3D7 and FC27)^24,25^. Recombinant *Pf*MSP2 has been characterized as highly disordered, but is known to form amyloid-like fibrils *in vitro*^26–30^. The propensity of *Pf*MSP2 to form these fibrillar structures has been linked to the key residues within the conserved N-terminal domain, but the fibril structure and rate of fibril formation may be modulated by CVR elements^26,29,31,32^. The exact structure of *Pf*MSP2 on the surface of the merozoite is unknown. However, recent evidence suggests that native *Pf*MSP2 may be conformationally constrained on the parasite membrane due to oligomerization^30^ and/or interaction with lipids^33–35^.

*Pf*MSP2-specific antibody responses elicited during natural infection have been correlated with acquired immunity in endemic populations, making *Pf*MSP2 an attractive vaccine candidate^7,36,37^. Furthermore, clinical trials of a *Pf*MSP2-containing multi-component vaccine demonstrated that induced anti-*Pf*MSP2 antibodies contribute to protective efficacy in an allele-specific manner^38,39^. Naturally-acquired and vaccine-induced anti-*Pf*MSP2 specific antibodies are primarily of the IgG1 and IgG3 isotypes and can function by complement-mediated mechanisms, or by cooperation with Fc receptor-bearing innate immune cells via antibody-dependent cellular inhibition (ADCI) and/or opsonic-phagocytosis^40–44^. As with other *P. falciparum* vaccine candidate antigens, success with *Pf*MSP2 vaccines has been limited by challenges linked to antigenic polymorphism, recombinant protein structure and immunogenicity. In an effort to address these challenges, we generated r*Pf*MSP2 and chimeric r*Pf*MSP2/*Pf*MSP8 (r*Pf*MSP2/8) vaccine antigens. Through comparative studies, we evaluated the ability of the *Pf*MSP8 carrier to affect yield, structure and/or immunogenicity of *Pf*MSP2 and the functionality of *Pf*MSP2-specific antibodies.

## Results

### Design, expression, and purification of unfused r*Pf*MSP2 and chimeric r*Pf*MSP2/8

**Figure 1:**
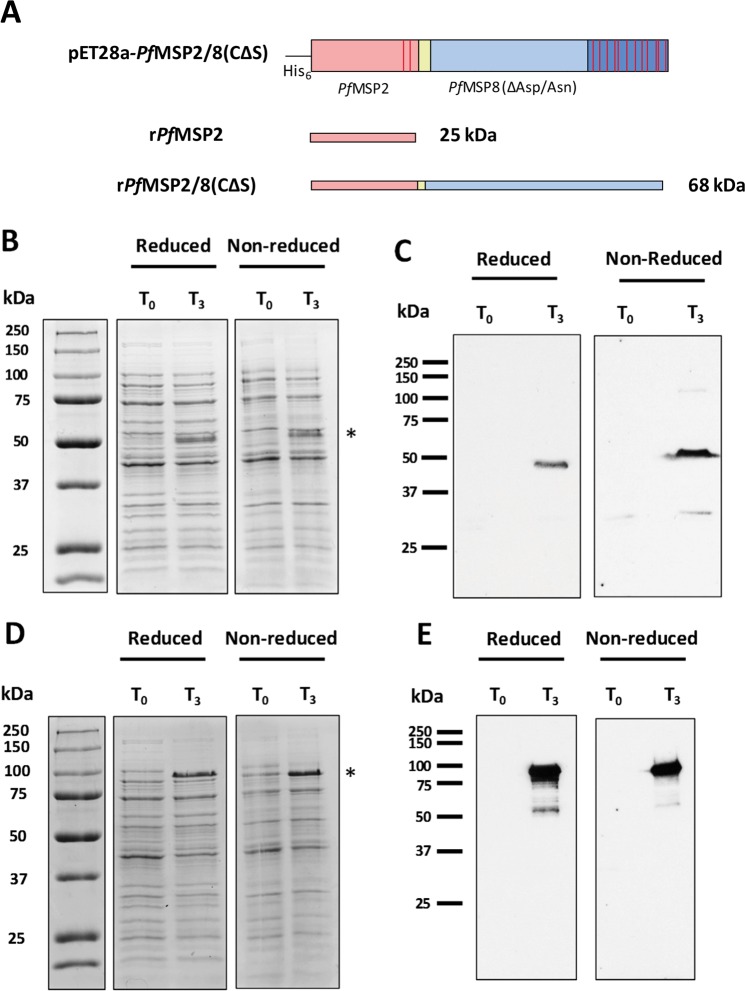
Expression of unfused r*Pf*MSP2 and chimeric r*Pf*MSP2/8. (**A**) Cartoon of expression constructs for r*Pf*MSP2 and r*Pf*MSP2/8 production. Cysteine residues are indicated by red bars, yellow region indicates Gly-Ser linker domain. (**B**,**C**) *E. coli* lysate at time of induction (T_0_) or 3 hours post-induction (T_3_) was separated on 10% polyacrylamide gels and r*Pf*MSP2 expression was analyzed by **(B**) Coomassie Blue staining and (**C**) immunoblot using anti-*Pf*MSP2 antibody (1:20,000 dilution). (**D**,**E**) r*Pf*MSP2/8 expression was analyzed by (**D**) Coomassie Blue staining and (**E**) immunoblot as described for r*Pf*MSP2. Asterisks indicate induced r*Pf*MSP2 and r*Pf*MSP2/8 protein products. The T_0_ samples served as negative controls for the immunoblot analysis of protein expression.

The mature coding sequence of the 3D7 allele of the *pfmsp2* gene was codon harmonized for expression in *E. coli* using previously established algorithms^45^. The codon harmonized synthetic gene was directionally subcloned into the pET28-MCS-*Pf*MSP8(CΔS) expression vector to facilitate production of two recombinant *Pf*MSP2-based antigens: an unfused r*Pf*MSP2 antigen (25 kDa) and a chimeric r*Pf*MSP2/8 antigen (68 kDa, Fig. 1A). Two stop codons were incorporated 3′ of the *Pf*MSP2 (3D7) coding sequence to generate the unfused construct. Following sequence verification, plasmids were transformed into SHuffle T7 Express *lysY E. coli* cells for expression. Pre- and post-induction samples of bacterial lysate were assessed for recombinant protein production via SDS-PAGE under reducing and non-reducing conditions. Coomassie Blue staining demonstrated robust protein expression of unfused r*Pf*MSP2 and chimeric r*Pf*MSP2/8 three hours post-induction (T_3_, Fig. 1B,D). Unfused r*Pf*MSP2 migrated as an ~50 kDa band, well above its predicted molecular weight of 24,721 daltons. This is consistent with previous reports documenting the aberrant migration of *Pf*MSP2^30^, attributed to its lack of hydrophobic residues. Anomalous migration during SDS-PAGE has been observed with several plasmodial proteins with disordered regions, highly charged repeat domains and/or deficits in hydrophobic residues^46,47^. Likewise, the chimeric r*Pf*MSP2/8 migrated as a corresponding ~100 kDa band, well above its predicted molecular weight of 67,659 daltons. Immunoblot analysis using polyclonal rabbit anti-*Pf*MSP2 sera confirmed the identity of r*Pf*MSP2 and r*Pf*MSP2/8 (Fig. 1C,E).

**Figure 2:**
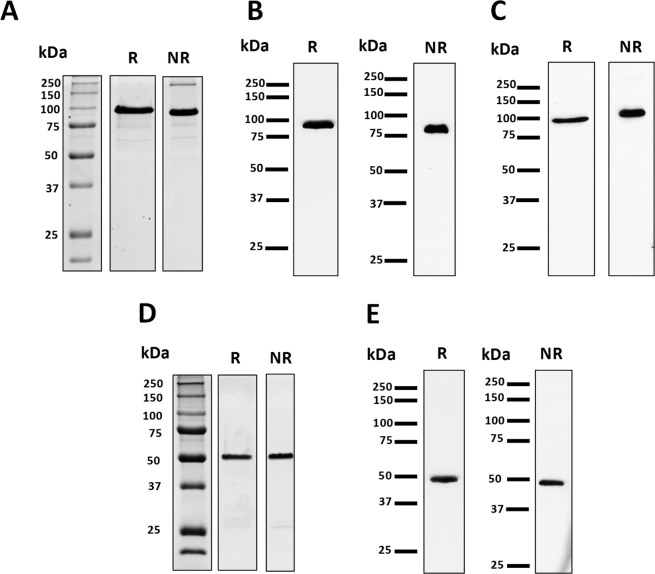
Purification of recombinant *Pf*MSP2-based antigens. (**A**–**C**) Analysis of purified r*Pf*MSP2/8 under reducing (R) and non-reducing (NR) conditions via (**A**) Coomassie Blue staining (3 μg/lane) or immunoblot (50 ng/lane) using **(B**) anti-*Pf*MSP2 antibody (1:5,000 dilution) or (**C**) anti-*Pf*MSP8 IgG (1 μg/ml). (**D**,**E**) Purified r*Pf*MSP2 was analyzed by (**D**) Coomassie Blue staining (3 μg/lane) or (**E**) immunoblot using anti-*Pf*MSP2 (100 ng/lane) as described for *Pf*MSP2/8.

Chimeric r*Pf*MSP2/8 was released from a bacterial inclusion body fraction by treatment with 0.2% sarkosyl and purified by nickel-chelate affinity chromatography under non-denaturing conditions with a final yield of 7.81 mg/g (wet weight) of bacterial cells. Purity was assessed via Coomassie Blue staining of 10% polyacrylamide gels run under reducing and non-reducing conditions, identifying the predominant product migrating at the expected ~100 kDa (Fig. 2A), with only a minor high molecular weight product observed under non-reducing conditions. Immunoblot analysis demonstrated strong reactivity of r*Pf*MSP2/8 with both rabbit anti-*Pf*MSP2 sera and rabbit anti-MSP8 IgG (Fig. 2B,C).

A similar procedure for lysis and purification was used for unfused r*Pf*MSP2. However, without the *Pf*MSP8 domain, the protein was present in the soluble supernatant following initial lysis. The soluble bacterial lysate was fractionated by ammonium sulfate precipitation prior to purification by nickel-chelate affinity chromatography under non-denaturing conditions. The final yield of unfused r*Pf*MSP2 was 4.22 mg/g (wet weight) of bacterial cells. Coomassie Blue staining of purified product under reducing and non-reducing conditions revealed a single, predominant protein migrating at the expected ~50 kDa (Fig. 2D). Strong reactivity with rabbit anti-*Pf*MSP2 sera was confirmed via immunoblot (Fig. 2E).

### r*Pf*MSP2, but not r*Pf*MSP2/8, forms amyloid-like fibrils *in vitro* which stimulate immune cells via TLR2

**Figure 3:**
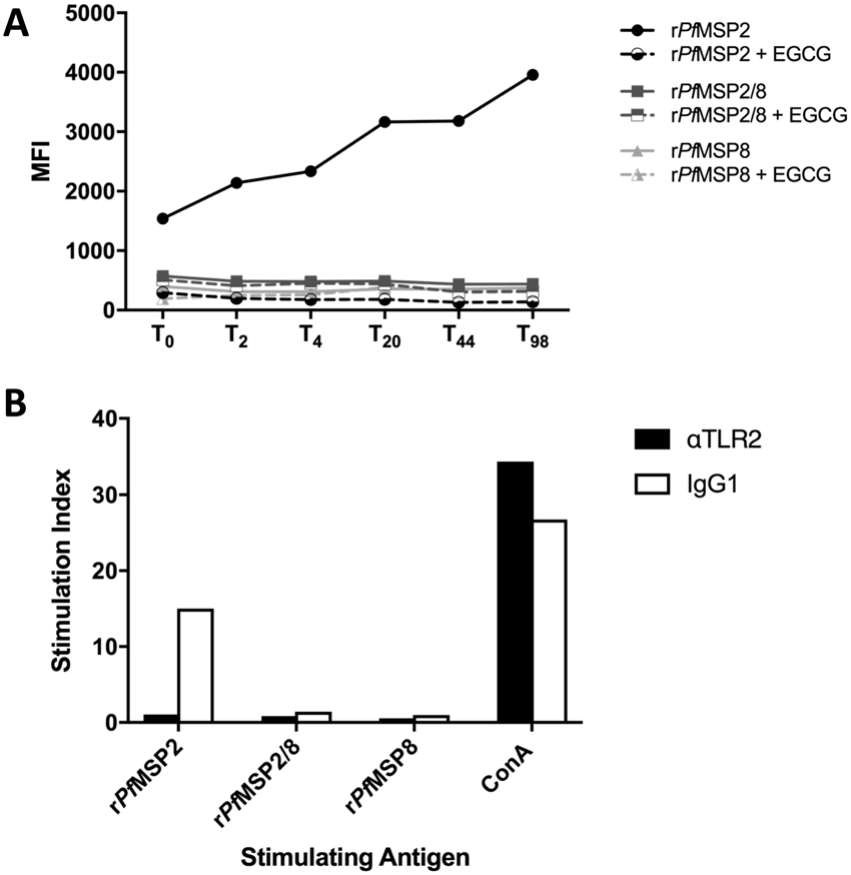
Assessment of fibril formation and stimulation of splenocytes with r*Pf*MSP2 antigens. (**A**) Recombinant proteins (10 μM) were stained with ThT (30 μM) and incubated at 37 °C. Fluorescence was measured at baseline (T_0_) prior to incubation and at indicated time points throughout (T_2_-T_98_, solid lines). In parallel, EGCG (40 μM) was co-incubated with recombinant proteins to prevent fibril formation and measured as above (dashed lines). Data are presented as the average of duplicate samples. (**B**) Naïve mouse splenocytes were plated, and triplicate samples were stimulated with recombinant antigen (10 μg/ml) in the presence of 1 μg/ml neutralizing anti-TLR2 mAb (black bars) or an IgG1 isoptype control (white bars). Concanavalin A (ConA, 1 μg/ml) served as positive control. Proliferation was quantitated by [^3^H]thymidine incorporation, and stimulation index was calculated for each sample relative to unstimulated controls.

It has been established that recombinant *Pf*MSP2 can form amyloid-like fibrils *in vitro*^26,29,48^. In an effort to minimize fibril formation, purified r*Pf*MSP2 and chimeric r*Pf*MSP2/8 were stored at −80 °C in buffer containing 0.2% sarkosyl. To evaluate fibrillar content immediately upon thawing, r*Pf*MSP2 and r*Pf*MSP2/8 were analyzed by size exclusion chromatography (SEC). Similar to previous studies^30,49^, recombinant r*Pf*MSP2 eluted as a prominent monomeric species at 3–4 times its predicted molecular weight. A second heterogeneous mixture of larger oligomeric species of r*Pf*MSP2 was also observed representing 5–10% of the total preparation (Fig. S1). In contrast, SEC analysis of r*Pf*MSP2/8 revealed single prominent monomeric protein (Fig. S1). To further assess the propensity of r*Pf*MSP2-based proteins to form fibrils, and to determine if genetic fusion of r*Pf*MSP2 to r*Pf*MSP8 affects fibril formation, a standard fluorescence-based assay was used. Thioflavin T (ThT), a stain that detects amyloid-like fibrils, was used to assess fibril formation of unfused r*Pf*MSP2, chimeric r*Pf*MSP2/8, or r*Pf*MSP8 carrier alone. Recombinant proteins were diluted in PBS without sarkosyl, prior to ThT staining and incubation at 37 °C for up to 98 hours. As shown in Fig. 3A, staining revealed fibrillar structures present in unfused r*Pf*MSP2 at baseline to a greater extent than in both r*Pf*MSP2/8 or r*Pf*MSP8. Fibril formation of the unfused r*Pf*MSP2 steadily increased during the incubation period, while the structure of chimeric r*Pf*MSP2/8 or r*Pf*MSP8 did not change relative to baseline. To further confirm the formation of amyloid-like fibrils, the assay was conducted in the presence of epigallocatechin-3-gallate (EGCG), an established inhibitor of fibril formation^27,28^. EGCG disrupted the fibrils present in the initial unfused r*Pf*MSP2 sample and inhibited additional fibril formation over time (Fig. 3A). Consistently, no difference in fibrillar content of r*Pf*MSP2/8 or r*Pf*MSP8 was observed. These data indicate that genetic fusion of r*Pf*MSP2 to r*Pf*MSP8 prevents the amyloid-like fibril formation characteristic of unfused r*Pf*MSP2.

Several fibrillar proteins have been shown to stimulate immune cells through TLR2. Therefore, to independently validate the differences in antigen structure determined above, the ability of fibrillar r*Pf*MSP2 versus non-fibrillar r*Pf*MSP2/8 or r*Pf*MSP8 to stimulate the proliferation of naïve mouse splenocytes was measured *in vitro*. A single cell suspension of splenocytes from a naïve CB6F1/J mouse was stimulated with 10 μg/ml of recombinant antigen in the presence of an anti-TLR2-blocking antibody or isotype control. Concanavalin A (ConA) was used as positive control for proliferation. As shown in Fig. 3B, r*Pf*MSP2 stimulated robust proliferation of splenocytes which was inhibited in the presence of the TLR2-blocking monoclonal antibody. r*Pf*MSP2/8 and r*Pf*MSP8, lacking fibrils, did not stimulate splenocytes to proliferate. Immunophenotyping experiments of naïve mouse splenocytes confirmed that among adaptive immune cell populations with proliferative capacity, B cells were the primary TLR2^+^ cell population with small populations of TLR2^+^ cells present among CD4^+^ and CD8^+^ T cells. Innate immune cell populations with limited proliferative capacity including dendritic cells, monocytes/macrophages and neutrophils represented only a minor population of TLR2^+^ splenocytes (data not shown). Together, these data demonstrate that fibrillar r*Pf*MSP2, but not r*Pf*MSP2/8, can stimulate naïve mouse splenocytes in a TLR2-dependent, antigen receptor-independent manner.

### Immunization with r*Pf*MSP2-based vaccines elicits high-titer, polyclonal antibody capable of recognizing native *P. falciparum* antigen

**Figure 4:**
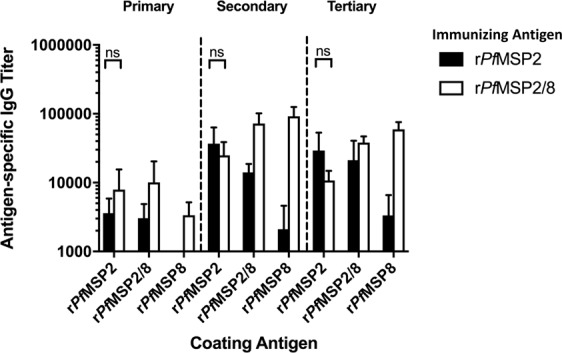
Immunization with r*Pf*MSP2-based vaccines elicits high-titer *Pf*MSP2-specific antibodies. New Zealand White (NZW) rabbits were immunized three times with r*Pf*MSP2 (*n* = 4, black bars) or r*Pf*MSP2/8 (*n* = 4, white bars) formulated with Montanide ISA 720. Antigen-specific IgG titers (mean ± SD) following each immunization were analyzed by ELISA. Signal detected by sera from the adjuvant control rabbit (*n* = 1) was subtracted from individual titers as background (Friedman test with Dunn’s multiple comparisons post hoc test; ns, not significant).

To generate high-titer, polyclonal rabbit sera, New Zealand White rabbits were immunized with unfused r*Pf*MSP2 (n = 4) or chimeric r*Pf*MSP2/8 (n = 4) antigen formulated with Montanide ISA 720, or with adjuvant alone (n = 1). Rabbits received three immunizations with serum samples collected 3 weeks following the primary and secondary immunizations, and with terminal bleed four weeks post tertiary immunization. Antigen-specific antibody titers were measured against each component antigen (r*Pf*MSP2, r*Pf*MSP2/8, or r*Pf*MSP8) by ELISA (Fig. 4). A single immunization with r*Pf*MSP2 or r*Pf*MSP2/8 elicited antibodies that bound to both r*Pf*MSP2- and r*Pf*MSP2/8- coated wells. Additionally, rabbits immunized with chimeric r*Pf*MSP2/8 generated a response against the r*Pf*MSP8 carrier domain, as expected. Following secondary immunization, antigen-specific antibody titers were boosted (anti-*Pf*MSP2 titers, *p* = 0.04; anti-*Pf*MSP8 titers, *p* < 0.02) to high levels but titers did not significantly increase further with a third immunization. Rabbits immunized with unfused r*Pf*MSP2 demonstrate low, but detectable, reactivity against r*Pf*MSP8 plate-bound antigen, which is potentially attributable to a shared epitope in a linker domain. Overall, high and comparable *Pf*MSP2-specific antibody titers were generated by immunization with r*Pf*MSP2 and r*Pf*MSP2/8.

**Figure 5:**
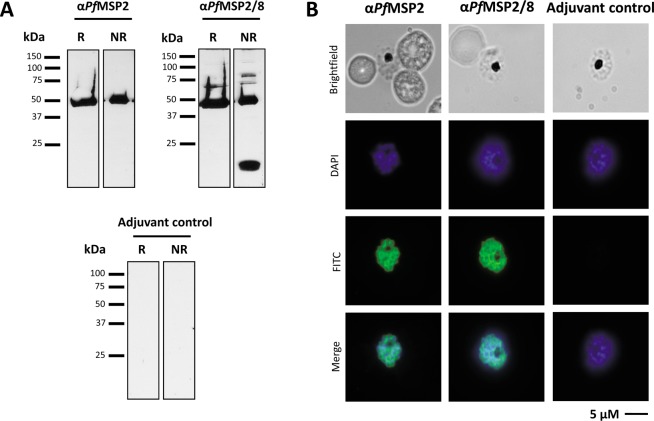
r*Pf*MSP2 and r*Pf*MSP2/8 vaccines induce antibody that recognizes native *P. falciparum* antigen. (**A**) Pools of anti-*Pf*MSP2 and anti-*Pf*MSP2/8 rabbit sera were used to determine recognition of native *P. falciparum* antigen via immunoblot of parasite lysate. (**B**) Detection of parasite-associated antigen using pooled sera was determined via indirect immunofluorescence assay of fixed, late-stage schizonts. Adjuvant control sera were also tested in both assays, serving as negative controls (R, reduced; NR, non-reduced).

The ability of antisera generated by immunization with r*Pf*MSP2-based vaccines to recognize native parasite protein was assessed via immunoblot and indirect immunofluorescence assay (IFA). A lysate of mixed *P. falciparum* blood-stage parasites was separated by SDS-PAGE, then blotted and probed with sera from r*Pf*MSP2- or r*Pf*MSP2/8- immunized rabbits, or adjuvant control sera. Strong recognition of parasite-derived *Pf*MSP2 was demonstrated using pooled sera from r*Pf*MSP2- and r*Pf*MSP2/8-immunized animals at the expected ~50 kDa when assessed under reducing and non-reducing conditions. Sera from animals immunized with r*Pf*MSP2/8 also recognized full-length native *Pf*MSP8 (~80 kDa) and the prominent ~17 kDa C-terminal processed product under non-reducing conditions only. As previously reported, anti-r*Pf*MSP8 antibodies are primarily conformation-dependent, directed against epitopes within the double EGF-like domain of its C-terminus^15^ (Fig. 5A). Immunoreactive proteins were not detected using adjuvant control sera. Pooled sera were also evaluated by indirect IFA of fixed *P. falciparum* late-stage schizonts. Sera from rabbits immunized with r*Pf*MSP2 and r*Pf*MSP2/8 demonstrated strong recognition of parasite-associated *Pf*MSP2 as noted by clear perimeter staining of the merozoite (Fig. 5B). No signal was detected on slides probed with adjuvant control sera (Fig. 5B, right). These data indicate that immunization with fibrillar r*Pf*MSP2 or non-fibrillar r*Pf*MSP2/8 generates high-titer *Pf*MSP2-specific antibodies that strongly recognize native *Pf*MSP2.

### *Pf*MSP2-based vaccine-induced antibodies recognize 3D7 and FC27 allelic families

**Figure 6:**
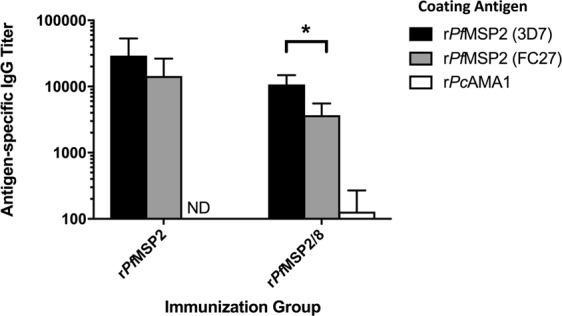
*Pf*MSP2 and *Pf*MSP2/8 vaccines induce r*Pf*MSP2 (3D7)- and r*Pf*MSP2 (FC27)- specific IgG. Antibody titers for r*Pf*MSP2 (3D7) or (FC27) allelic families were determined by ELISA (mean ± SD) using tertiary sera from each rabbit. r*Pc*AMA1-coated wells were used to measure His_6_-specific IgG. Adjuvant control responses were subtracted from individual titers as background. Asterisk indicates significance (Mann-Whitney U test, *p* < 0.03).

To quantitatively assess the levels of allele-specific and cross-reactive antibodies induced by r*Pf*MSP2 (3D7)-based vaccines, recombinant *Pf*MSP2 (FC27) was generated. The mature coding sequence of the FC27 allele of the *pfmsp2* gene was codon harmonized for expression in *E. coli*, synthesized, subcloned and expressed as described above for r*Pf*MSP2 (3D7). During purification, an additional denaturation and glutathione-catalyzed renaturation procedure was necessary to eliminate aggregates of r*Pf*MSP2 (FC27) and promote proper disulfide bond formation. To assess potential differences in cross-reactive antibody populations generated by r*Pf*MSP2 and r*Pf*MSP2/8, antibodies capable of binding r*Pf*MSP2 (3D7) or r*Pf*MSP2 (FC27) were quantified by ELISA. Sera from animals immunized with r*Pf*MSP2 (3D7) were equally reactive with plated-bound r*Pf*MSP2 (3D7) and r*Pf*MSP2 (FC27) with no significant difference in IgG titers (*p* = 0.20). While immunization with r*Pf*MSP2/8 (3D7) induced antibodies that recognized the two major alleles of *Pf*MSP2, titers against the homologous r*Pf*MSP2 (3D7) were significantly higher than titers against the heterologous r*Pf*MSP2 (FC27) (*p* < 0.03, Fig. 6). Antibody binding to the shared His_6_ -tag was minimal as assessed by binding to recombinant *P. chabaudi* AMA1, an unrelated, but His_6_ -tagged control antigen. These data suggest that there are differences in the fine-specificity of antibodies elicited by immunization with r*Pf*MSP2 versus r*Pf*MSP2/8.

### Differences in antibody populations induced by fibrillar vs non-fibrillar *Pf*MSP2-based vaccine antigens

**Figure 7:**
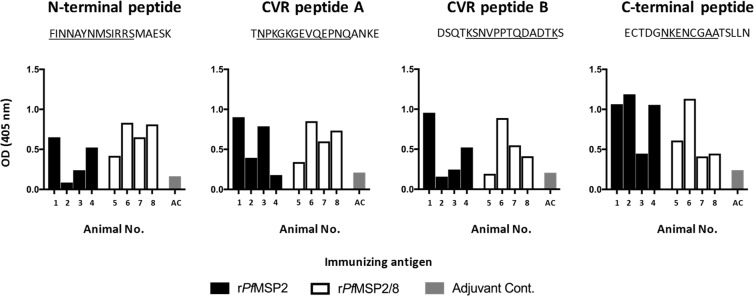
r*Pf*MSP2-based vaccines induce antibodies that recognize established mAb epitopes. To determine the impact of antigen conformation on antibodies induced by immunization, *Pf*MSP2 peptides containing established mAb epitopes were used for analysis via ELISA. Sera from individual immunized rabbits (black, white) and control rabbit (grey) were diluted and tested for reactivity with each peptide. Data are presented as the average of duplicate A_405_ values. Underlined portion of peptide sequence represents mAb epitopes 6D8, 11E1, 9D11 and 4D11/9G8 (left to right).

Fusion of r*Pf*MSP2 to r*Pf*MSP2/8 affected the structure of r*Pf*MSP2 by eliminating the propensity for fibrillization (Fig. 3). To begin to assess the impact of such conformational changes on antibody epitope specificity, the reactivity of vaccine-induced antibodies to known B cell epitopes of *Pf*MSP2 was profiled by ELISA. The four *Pf*MSP2 peptides (18-mers) evaluated contained i) the conserved N-terminal region epitope recognized by mAb 6D8, ii) two *Pf*MSP2 (3D7)-specific CVR epitopes recognized by mAb 11E1 and mAb 9D11, respectively, and iii) the conserved C-terminal region epitope recognized by mAb 4D11 and 9G8^48^. As shown in Fig. 7, sera from animals immunized with fibrillar r*Pf*MSP2 recognized the N-terminal epitope and the two CVR epitopes to varying degrees, with stronger recognition of the C-terminal epitope. Sera from animals immunized with non-fibrillar *rPf*MSP2/8 recognized all four epitopes, but the response was more uniformly distributed across epitopes in comparison to r*Pf*MSP2-induced sera. These data are consistent with a more open conformation of the r*Pf*MSP2 molecule when fused to the r*Pf*MSP8 carrier. Of significance, sera from all four r*Pf*MSP2/8-immunized rabbits recognized the conserved C-terminal epitope, confirming that fusion of *Pf*MSP8 to the C-terminus of *Pf*MSP2 did not alter epitope accessibility near this junction (Fig. 7, right panel).

To further evaluate differences in the nature of the *Pf*MSP2-specific epitopes recognized by antibodies induced by immunization with fibrillar r*Pf*MSP2 or non-fibrillar r*Pf*MSP2/8, seroreactivity was evaluated by competition ELISA. The ability of r*Pf*MSP2 (3D7) (fibrillar) and r*Pf*MSP2/8 (non-fibrillar) to inhibit binding of anti-*Pf*MSP2 (3D7) or anti-*Pf*MSP2/8 antibody to plate-bound immunizing antigen was quantified. To eliminate the detection of *Pf*MSP8-specific antibody binding, 1 μM of r*Pf*MSP8 was included in all antibody + inhibitor incubations. At this concentration of r*Pf*MSP8, minimal residual binding was detected against plate-bound *Pf*MSP8.

**Figure 8:**
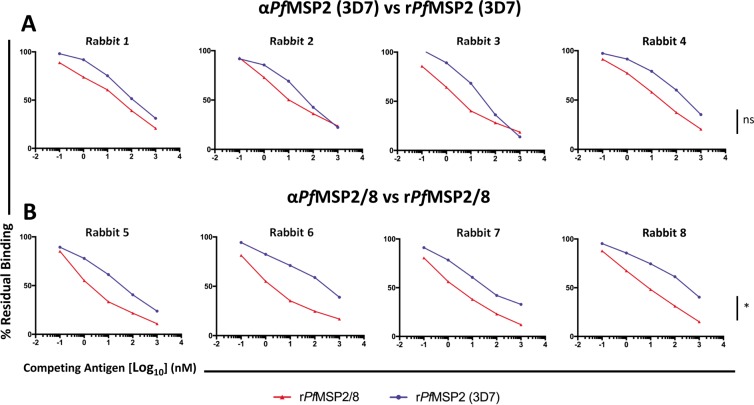
r*Pf*MSP2-associated fibrils mask relevant *Pf*MSP2-specific epitopes. Competition ELISAs were conducted using a fixed concentration of rabbit antibody induced by (**A**) r*Pf*MSP2 or (**B**) r*Pf*MSP2/8 immunization. Sera were co-incubated in solution with increasing amounts of r*Pf*MSP2 or r*Pf*MSP2/8. Residual binding to plate-bound immunizing antigen for each antigen + antibody cocktail was measured in duplicate by ELISA and calculated as (A_405_ of IgG with inhibitor/A_405_ without inhibitor) × 100. Statistical significance was conducted at maximum levels of inhibition. Data from samples within an immunization group were averaged and compared. Asterisk indicates significance (Mann-Whitney U test, *p* < 0.03; ns, not significant).

In the first set of assays, the ability of r*Pf*MSP2 (3D7) or r*Pf*MSP2/8 to inhibit the binding of anti-*Pf*MSP2 (3D7) sera to plate-bound r*Pf*MSP2 (3D7) was tested. r*Pf*MSP2 (3D7) efficiently inhibited a high proportion of the anti-*Pf*MSP2 (3D7) antibody binding with 65–86% inhibition at the highest concentrations of inhibitor (Fig. 8A, blue). Chimeric r*Pf*MSP2/8 was equally as effective at inhibiting anti-*Pf*MSP2 antibody binding to plate-bound *Pf*MSP2 (3D7) with 76–81% inhibition at the highest concentrations (Fig. 8A, red; Table 1, *p* > 0.48). The second set of assays tested the ability of r*Pf*MSP2 (3D7) or r*Pf*MSP2/8 to inhibit the binding of anti-*Pf*MSP2/8 sera to plate bound r*Pf*MSP2/8. Chimeric r*Pf*MSP2/8 was shown to be a potent inhibitor of anti-*Pf*MSP2/8 antibody binding with 83–90% inhibition at high concentrations of inhibitor (Fig. 8B, red). Comparatively, r*Pf*MSP2 (3D7) inhibited anti-*Pf*MSP2/8 antibody binding to plate-bound r*Pf*MSP2/8 (60–79% inhibition) to a significantly lesser degree than r*Pf*MSP2/8, even at the highest concentrations tested (Fig. 8A, blue; Table 1, *p* < 0.03). Combined, these data indicate that antibodies generated by immunization with chimeric r*Pf*MSP2/8 consistently recognize a broad range of *Pf*MSP2 epitopes that are displayed on r*Pf*MSP2/8, but are presumably masked on unfused r*Pf*MSP2 due to its fibrillar nature (Table 1).

**Table 1:** Anti-*Pf*MSP2/8 IgG recognizes PfMSP2-specific epitopes masked by r*Pf*MSP2-associated fibrils.

Rabbit Sera	Inhibitor	Maximum % Inhibition (Mean ± SD)	*p* value
α*Pf*MSP2 (3D7)	r*Pf*MSP2/8	79.1 ± 2.1	0.4857
	r*Pf*MSP2 (3D7)	74.4 ± 9.6	
α*Pf*MSP2/8	r*Pf*MSP2/8	86.3 ± 2.7	0.0286
	r*Pf*MSP2 (3D7)	66.0 ± 7.5	

### Functional activity of antibodies induced by immunization with r*Pf*MSP2 versus r*Pf*MSP2/8

The growth inhibition assay (GIA) was used to determine the ability of antibodies induced by immunization with r*Pf*MSP2-based vaccines to inhibit *in vitro* growth of blood-stage *P. falciparum* parasites. Purified IgG from each rabbit was tested at a concentration of 2.5 mg/ml and 5.0 mg/ml. At 2.5 mg/ml, there was no detectable growth inhibitory activity in any of the four anti-*Pf*MSP2 IgG samples (Table 2). When the IgG concentration was elevated to 5.0 mg/ml, a low level of growth inhibition was observed in three out of four samples. Similarly, all four anti-*Pf*MSP2/8 IgG samples demonstrated no growth inhibitory activity at 2.5 mg/ml. This remained consistent for three out of four samples when the IgG concentration was raised to 5.0 mg/ml (Table 2). The low and variable growth inhibitory activity for anti-*Pf*MSP2 and anti-*Pf*MSP2/8 samples at high concentration were comparable to adjuvant control background levels. As anticipated, anti-*Pf*MSP1/8 IgG positive control samples demonstrated a high levels of growth inhibition in both assays with ~60% inhibition at 2.5 mg/ml and ~80% inhibition at 5.0 mg/ml. These data show that antibodies induced by immunization with r*Pf*MSP2 or r*Pf*MSP2/8 are not functional in this assay. These data are consistent with previously reported GIA data for naturally-acquired and vaccine-induced *Pf*MSP2-specific antibodies^50–52^.

**Table 2:** *In vitro* inhibition of *P. falciparum* (NF54) by rabbit MSP-specific IgG.

Assay	Rabbit antiserum	% Growth Inhibition	
		2.5 mg/ml	5 mg/ml
	(animal no.)	IgG	IgG
1	*Pf*MSP2 (1)	ND	15
	*Pf*MSP2 (2)	ND	ND
	*Pf*MSP2 (3)	ND	3
	*Pf*MSP2 (4)	ND	2
	Adjuvant Control	ND	10
	*Pf*MSP1/8 Control	58	80
2	*Pf*MSP2/8 (5)	ND	ND
	*Pf*MSP2/8 (6)	ND	ND
	*Pf*MSP2/8 (7)	ND	ND
	*Pf*MSP2/8 (8)	ND	19
	Adjuvant Control	2	4
	*Pf*MSP1/8 Control	61	79

ND, not detected.

**Figure 9:**
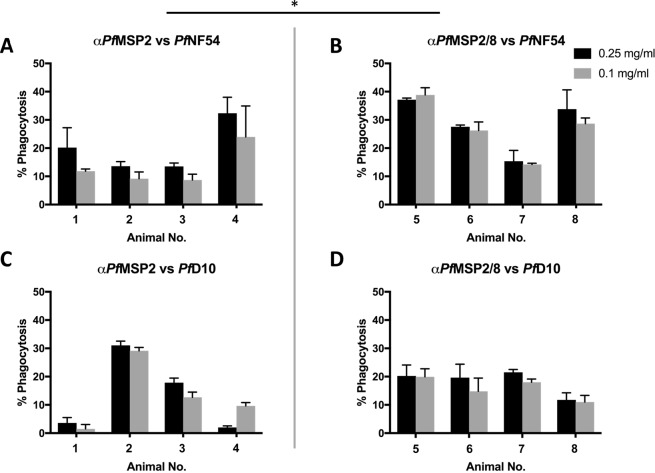
Anti-*Pf*MSP2/8 IgG exhibits enhanced functionality in the opsonophagocytosis assay as compared to anti-*Pf*MSP2. (**A**,**B**) The ability of purified (**A**) *Pf*MSP2 IgG or (**B**) *Pf*MSP2/8 IgG to enhance phagocytosis of homologous *P. falciparum* merozoites was measured by OPA at high (0.25 mg/ml, black) and low (0.1 mg/ml, grey) concentrations. (**C**,**D**) Parallel assays with (**C**) *Pf*MSP2 IgG and (**D**) *Pf*MSP2/8 IgG were performed on heterologous merozoites. Phagocytosis was tested in triplicate and quantified as the percent fluorescent THP-1 following co-incubation with opsonized merozoites (% phagocytosis, mean ± SD). Concentration-matched adjuvant control-opsonized samples were subtracted as background. For statistical analysis, data from individual animals within an immunization group were averaged and compared. Asterisk indicates significance (Mann-Whitney U test, *p* < 0.05).

The functionality of *Pf*MSP2-specific antibodies, in cooperation with monocytes/macrophages, has been previously demonstrated using an *in vitro* opsonophagocytosis assay (OPA)^43^. To test the opsonizing capacity of anti-*Pf*MSP2 and anti-*Pf*MSP2/8 antibodies, an adapted and optimized OPA was used. Purified IgG from each animal was tested for the ability to opsonize homologous (*P. falciparum* NF54) and heterologous (*P. falciparum* D10) merozoites for phagocytosis by THP-1 monocytic cells. Prior to use, the sequence of the *msp2* gene of the NF54 and D10 strains of *P. falciparum* was confirmed to be the 3D7 and FC27 *msp2* allelic variants, respectively. As shown in Fig. 9A, phagocytosis of *P. falciparum* NF54 merozoites opsonized with anti-*Pf*MSP2 IgG at a concentration of 0.1 mg/ml ranged only from 9–24%. A modest increase to 14–32% phagocytosis was observed when the concentration was raised to 0.25 mg/ml. At a concentration of 0.1 mg/ml, opsonization with anti-*Pf*MSP2/8 IgG promoted the efficient phagocytosis of homologous *P. falciparum* NF54 merozoites (14–39% phagocytosis), a significant increase relative to phagocytosis observed with anti-*Pf*MSP2 IgG at the same concentration of 0.1 mg/ml *(p* = 0.05). Comparable levels of phagocytosis were observed when the anti-*Pf*MSP2/8 IgG concentration was increased to 0.25 mg/ml (15–37% phagocytosis, Fig. 9B). Assessment of anti-*Pf*MSP8 IgG confirmed that increased phagocytic activity of r*Pf*MSP2/8 IgG was not due to reactivity of anti-*Pf*MSP8 antibodies (Table S1).

To evaluate the cross-strain neutralizing capacity of vaccine-induced antibodies, parallel OPAs were performed with merozoites isolated from the heterologous *P. falciparum* D10 strain. As shown in Fig. 9C, opsonization of heterologous merozoites with anti-*Pf*MSP2 IgG resulted in variable and generally low levels of phagocytosis (2–13% at 0.1 mg/ml and 4–18% phagocytosis at 0.25 mg/ml). The exception was one anti-*Pf*MSP2 IgG sample that led to ~30% phagocytosis at both concentrations (Fig. 9C). In contrast, opsonization of heterologous merozoites with anti-*Pf*MSP2/8 IgG promoted a modest level of phagocytosis of a concentration of 0.1 mg/ml (11–20%), with comparable levels at 0.25 mg/ml (12–20%, Fig. 9D). Opsonization of heterologous merozoites by anti-*Pf*MSP2 IgG and anti-*Pf*MSP2/8 IgG was not significantly different (*p* > 0.48). Together, these data showed that anti-*Pf*MSP2/8 IgGs were consistently and potently functional in their capacity to opsonize homologous and heterologous merozoites for phagocytosis by THP-1 cells. In contrast, anti-*Pf*MSP2 IgGs were less potent and more variable in their capacity to enhance phagocytosis of homologous merozoites.

## Discussion

We firmly believe that the development of a highly efficacious, multi-stage vaccine will be necessary to achieve the goals of malaria elimination and eradication. We also believe that an essential component of that vaccine will need to target blood-stage malaria parasites. The complexity of the host immune response to merozoites, the low immunogenicity of vaccine candidates, and the limited availability of potent, human-compatible adjuvants, are significant challenges for the vaccine effort. For subunit malaria vaccines, various vaccine platforms, immunization strategies and potential candidate antigens have been evaluated, but progress has been slow. Our lab has focused on the use of a *Plasmodium*-specific carrier protein, *Pf*MSP8, to enhance production and immunogenicity of leading vaccine antigens^15^. We have validated this approach with the genetic fusion of *Pf*MSP1_19_ and *Pf*s25 to the *Pf*MSP8 carrier protein. With chimeric r*Pf*MSP1/8 and r*Pf*s25/8 antigens, overall production and immunogenicity was improved while the conformation of critical protective epitopes and domains was retained. Antibody responses against the *Pf*MSP1_19_ and *Pf*s25 targeted domains were strong, and the elicited IgGs were highly functional in growth inhibition and standard membrane-feeding assays, respectively^16–18^. These results provided a clear rationale to evaluate another leading vaccine candidate, *Pf*MSP2, with *Pf*MSP8 as its carrier.

*Pf*MSP2 is an attractive blood-stage vaccine candidate. *Pf*MSP2 elicits an immune response in *P. falciparum* infected individuals living in endemic areas that has been correlated with protection^36,37^. This protective antibody response has been characterized as allele-specific, suggesting that the central variable repeat regions are immunodominant in the context of infection^48,53^. In Phase IIb trials of the Combination B vaccine, anti-MSP2-specific responses contributed to vaccine efficacy^38,39^. Again, protection was *Pf*MSP2-allele specific suggesting that a successful *Pf*MSP2-containing vaccine may need to include both major alleles. If possible for a *Pf*MSP2-containing vaccine, however, induction of a protective, cross-reactive immune response targeting conserved N- and C- terminal *Pf*MSP2 epitopes with sufficient magnitude and durability would be highly desirable. Results from a recent study analyzing antibodies induced by a bi-allelic MSP2-based vaccine in Phase I trials exemplify this paradigm, demonstrating that co-immunization with both allelic families of PfMSP2 induced cross-reactive, functional antibodies^54^. As such, recent studies of *Pf*MSP2-based vaccines have focused on partial and/or 3D7-FC27 fusion proteins that exclude portions of the *Pf*MSP2 full-length protein in an effort to simplify production, eliminate fibrillar aggregates and/or enhance the antibody response to conserved B cell epitopes^55,56^. Some success has been achieved with long synthetic *Pf*MSP2 peptides and with the *Pf*MSP2 3D7-FC27 mosaics, which have demonstrated immunogenicity in mouse studies, as well as reactivity with sera from semi-immune adults living in malaria endemic areas^49,57^. However, there is inadequate information on the relationship between immunizing antigen and *Pf*MSP2 structure on the surface of the merozoite to adequately justify elimination of selected *Pf*MSP2 domains from vaccine constructs. It is important to keep in mind that although *Pf*MSP2 is characterized as a highly disordered protein, recent evidence indicates that *Pf*MSP2 on the merozoite surface may be conformationally constrained due to oligomerization or interaction with membrane lipids, in addition to the GPI anchor^21,33^. Additionally, the protective anti-*Pf*MSP2 antibody responses detected in individuals living in endemic areas primarily target readily accessible, allele-specific epitopes^36,37^.

In this study, we were able to produce chimeric r*Pf*MSP2/8 in high yield using straightforward purification protocols. We used size exclusion chromatography and a fluorescence-based assay to monitor the extent of fibril formation of r*Pf*MSP2/8 versus r*Pf*MSP2. Our results showed that unfused r*Pf*MSP2 formed fibrils, however, upon fusion to the *Pf*MSP8 carrier, r*Pf*MSP2 fibril formation was prevented. Of note, reactivity of *Pf*MSP2 (3D7)-associated fibrils with ThT has not previously been reported. Adda *et al*. have shown that r*Pf*MSP2 (FC27) fibrils can be detected by ThT fluorescence, but that r*Pf*MSP2 (3D7) fibrils do not bind the dye. The r*Pf*MSP2 (3D7) produced by Adda *et al*. contained a C-terminal His_6_ tag while our r*Pf*MSP2 (3D7) was tagged at its N-terminus. As such, the presence or absence of the His-tag at the N-terminus, where fibril formation has been proposed to initiate,^26,29,31,32^ may have influenced fibril formation and/or ThT binding to some degree. Further evidence of structural differences between r*Pf*MSP2 and r*Pf*MSP2/8 were obtained, as only r*Pf*MSP2 stimulated splenocyte proliferation through TLR2. Binding and signaling through TLR2 has been reported as a characteristic of some fibrillar proteins^58,59^. It is unclear how TLR2-mediated recognition of fibrillar *Pf*MSP2 impacts infection-induced immune responses elicited by native, parasite-associated *Pf*MSP2 *in vivo*, but it is of interest and will require further study.

Because of the uncertainty regarding the structure of native *Pf*MSP2 on the surface of the merozoite, we pursued an evaluation of the immune response to fibrillar versus non-fibrillar *Pf*MSP2 in an effort to inform r*Pf*MSP2-based vaccine design. We aimed to determine whether the fibrillar nature of r*Pf*MSP2 skewed the immune response toward the generation of cross-reactive or allele-specific antibodies. Upon examining the ability of r*Pf*MSP2 vaccine-induced antibody to recognize both allelic families of r*Pf*MSP2, we observed no significant difference in antibody titers when assayed via ELISA. However, anti-*Pf*MSP2/8 sera contained significantly higher titers of antibodies that recognized r*Pf*MSP2 (3D7) compared to r*Pf*MSP2 (FC27). It is possible that these results were influenced by the conformation of plate-bound, unfused r*Pf*MSP2 and the accessibility of B cell epitopes.

We determined that fusion of r*Pf*MSP2 to r*Pf*MSP8 carrier affects the conformation of the r*Pf*MSP2 component. This fusion could potentially alter accessibility of relevant B cell epitopes, particularly in the conserved C-terminal region of *Pf*MSP2 near the junction with *Pf*MSP8. However, antibody induced by immunization with r*Pf*MSP2/8 bound to a peptide containing a conserved C-terminal region epitope recognized by mAbs 4D11 and 9G8. These data confirm that this epitope was not altered or blocked by fusion of r*Pf*MSP2 to *Pf*MSP8. With this approach, we also profiled the reactivity to a conserved N-terminal epitope defined by mAb 6D8, and two 3D7-associated CVR epitopes recognized by mAbs 11E1 and 9D11. The results suggest that in contrast to r*Pf*MSP2, immunization with r*Pf*MSP2/8 induced a more balanced antibody response distributed across epitopes throughout r*Pf*MSP2.

Competitive-binding ELISAs provided a unique and effective opportunity to further parse out the potential differences in the fine specificity of r*Pf*MSP2- or r*Pf*MSP2/8- induced antibodies. In this series of competition assays, non-fibrillar antigen in an open and uniform conformation (r*Pf*MSP2/8) or antigen with a mixture of monomeric and fibrillar conformations (r*Pf*MSP2) were used in solution to compete out *Pf*MSP2-specific antibody binding to plate-bound immunizing antigen. These data identified a population of *Pf*MSP2-specific antibodies elicited by r*Pf*MSP2/8 immunization that could not be effectively competed out by homologous fibrillar r*Pf*MSP2. This was only observed in sera from r*Pf*MSP2/8-immunized animals, as both fibrillar and non-fibrillar *Pf*MSP2 efficiently inhibited binding of antibodies elicited by immunization with unfused r*Pf*MSP2. These results suggest that the r*Pf*MSP2/8 vaccine induced an additional population of antibodies that recognized *Pf*MSP2-specific epitopes masked by fibrils formed by the r*Pf*MSP2 antigen. Together, with the results from SEC, ThT fluorescence assays and TLR2-dependent splenocyte proliferation assays (Fig. 3, S1), we have established that r*Pf*MSP2 fibrillar content impacts the display of *Pf*MSP2 epitopes, and that the non-fibrillar conformation of r*Pf*MSP2/8 increases the extent to which *Pf*MSP2 epitopes are accessible.

Vaccine-induced *Pf*MSP2-specific antibodies that are cross-reactive between the two major allelic variants are desirable. However, it is critical that these antibodies are functional in the neutralization and clearance of merozoites. The GIA is an established assay to assess the functionality of antibodies to merozoite antigens. However, analyses of naturally-acquired and vaccine-induced *Pf*MSP2-specific antibodies have inconsistently demonstrated the ability to inhibit parasite growth in this assay. As such, we sought to determine if IgG induced by our *Pf*MSP2-based vaccines demonstrated *in vitro* growth inhibitory activity. We did not observe any significant growth inhibition by *Pf*MSP2-specific IgG derived from animals immunized with either chimeric r*Pf*MSP2/8 or unfused r*Pf*MSP2 vaccines. These results are consistent with previous studies of both vaccine-induced and naturally-acquired *Pf*MSP2-specific antibodies^50–52^.

Recent development and use of the opsonophagocytosis assay (OPA) has contributed to the understanding of anti-*Pf*MSP2 antibody functionality and has proven to be a better measure of *Pf*MSP2-dependent immunity than the GIA^43^. The ability of rabbit IgG (produced as a single isotype) to bind to human Fc receptors on THP-1 cells has previously been exploited for analysis of immunization-induced IgG in the OPA^60^. Therefore, to measure the functionality of our rabbit anti-*Pf*MSP2 antibodies, we utilized the OPA. Here, we simply report the percent of THP-1 cells containing fluorescent merozoites, allowing direct side-by side comparison of the opsonizing capacity of immunization-induced rabbit IgGs. This is in contrast to Beeson *et al*., who report relative phagocytic index (RPI) as a percentage of the test antibody activity relative to a positive control^43,54,60^. We demonstrated that anti-*Pf*MSP2/8 IgG effectively opsonized homologous merozoites (*P. falciparum* NF54) at both concentrations tested (0.1 and 0.25 mg/ml). Conversely, the functionality of anti-*Pf*MSP2 IgG was significantly lower than that of anti-*Pf*MSP2/8 IgG overall, and more variable between rabbits. Opsonization of heterologous merozoites (*P. falciparum* D10) and phagocytosis by THP-1 cells was comparable between antibodies elicited by immunization with r*Pf*MSP2 and r*Pf*MSP2/8, as supported by our evaluation of cross-reactive antibody responses (Fig. 6). Combined, the data indicate that antibodies induced by immunization with non-fibrillar, chimeric *Pf*MSP2/8 are able to recognize relevant *Pf*MSP2-specific epitopes that contribute to antibody functionality. Of interest, these *Pf*MSP2/8-induced antibodies were more potently functional against parasites expressing the homologous allele of *Pf*MSP2. Altogether, we conclude that r*Pf*MSP2 and r*Pf*MSP2/8 vaccines induce an equally potent amount of cross-reactive antibody, but that an additional population of *Pf*MSP2-specific antibodies induced by r*Pf*MSP2/8 are allele-specific and functional in the OPA.

There is still more to learn about the structural differences between *Pf*MSP2 on the surface of the merozoite and *Pf*MSP2 in purified, recombinant form. In the meantime, it is essential to evaluate the ability of vaccine-induced *Pf*MSP2-specific antibody not only to recognize parasite-associated antigen, but also to function in a way that facilitates merozoite neutralization. Through fusion to *Pf*MSP8, we have affected the structure and immunogenicity of r*Pf*MSP2 and increased accessibility of neutralizing epitopes on r*Pf*MSP2. We believe this is of significant benefit from a vaccine perspective. While unfused r*Pf*MSP2 is immunogenic, it exists as a mix of fibrillar and non-fibrillar structures that changes with time, temperature, pH, etc. This presents significant challenges for uniform production of a vaccine antigen that can elicit antibody responses of predictable specificity that can be easily measured in conventional immunoassays. These issues are markedly reduced with the chimeric r*Pf*MSP2/8. Our data also suggest that a bi-allelic *Pf*MSP2/8-based vaccine that can induce allele-specific, neutralizing antibodies to both *Pf*MSP2 variants, in addition to cross-strain neutralizing antibodies, may improve overall efficacy. Finally, these *Pf*MSP2 vaccine studies provide further support for the broad application of *Pf*MSP8 as a carrier for subunit malaria vaccine antigens.

## Methods

### Design and Production of unfused *Pf*MSP2 and chimeric *Pf*MSP2/8

The coding sequences of *pfmsp2* (3D7 allele, amino acids 21–248; FC27 allele, amino acids 21–240) were codon harmonized for expression in *E. coli* as previously described^45^. SpeI and AflII restriction sites were added to the 5′ and 3′ ends respectively, to facilitate directional cloning. The *Pf*MSP2 (3D7) codon harmonized gene (GenBank Accession # MK450589) and *Pf*MSP2 (FC27) codon harmonized gene (GenBank Accession # MK450590) were commercially synthesized (Blue Heron Biotechnology Inc., Bothell, WA). The synthetic *Pf*MSP2 (3D7) gene was then subcloned into pET28a-MCS-*Pf*MSP8(CΔS)^17^ for expression of chimeric *Pf*MSP2/8 (3D7). For expression of unfused *Pf*MSP2 (3D7) and *Pf*MSP2 (FC27), two stop codons (TGA, TAA) were incorporated at the 3′ end of the *Pf*MSP2 coding sequences. *Pf*MSP2 sequences were verified (GENEWIZ, South Plainfield, NJ) prior to transformation into SHuffle T7 express *lysY* competent *E. coli* cells (New England Biolabs, Ipswich, MA).

Bacteria were grown in enriched media (5 L) using a BioFlo115 Benchtop Bioreactor (New Brunswick Scientific) and recombinant antigens expressed as previously described^17^. Cells were harvested by centrifugation, washed in phosphate-buffered saline (PBS, pH 7.4) and stored as pellets at −80 °C. Protein expression was assessed by SDS-PAGE on 10% polyacrylamide gels followed by Coomassie Blue staining or immunoblot analysis using polyclonal rabbit antibody specific for *Pf*MSP2. This anti-*Pf*MSP2 serum was obtained through the Malaria Research and Reference Reagent Resource Center as part of the BEI Resources Repository, NIAID, NIH: *Plasmodium falciparum* Anti-MSP2 Antibodies, MRA-318, deposited by RL Coppel (MR4, BEI Resources, ATCC, Manassas, VA). Bound antibody was detected by horseradish-peroxidase (HRP)-conjugated goat anti-rabbit IgG (0.1 μg/ml; Thermo Fisher Scientific) and SuperSignal^TM^ West Pico chemiluminescent substrate (Thermo Fisher Scientific).

### Purification of *rPf*MSP2/8 and r*Pf*MSP2

To purify r*Pf*MSP2/8, bacterial pellets were lysed using BugBuster HT protein extraction reagent in the presence of recombinant lysozyme and benzonase nuclease (Millipore Sigma, St. Louis, MO). Inclusion bodies were pelleted, washed and solubilized in Binding Buffer (20 mM Tris-HCl, pH 7.9, 0.5 M NaCl, 5 mM imidazole) containing 0.2% sarkosyl (N-lauroylsarcosine, Millipore Sigma) at 4 °C, overnight with rotation. Residual insoluble material was pelleted at 7600 × g, and r*Pf*MSP2/8 was purified from the soluble fraction by nickel-chelate affinity chromatography (Ni-NTA agarose beads; Qiagen, Valencia, CA) under non-denaturing conditions in the presence of 0.2% sarkosyl. A portion of the r*Pf*MSP2/8 that remained in the unbound fraction was reapplied to the Ni-NTA column to maximize yield. The final r*Pf*MSP2/8 product was dialyzed into 25 mM Tris-HCl, pH 8.0, 150 mM NaCl (TBS) containing 0.2% sarkosyl. For purification of r*Pf*MSP2 (3D7 and FC27), bacterial pellets were lysed as above. Following centrifugation, the soluble supernatant containing r*Pf*MSP2 was fractionated by treatment with 20% ammonium sulfate. The resulting supernatant was dialyzed overnight at 4 °C into Binding Buffer +0.2% sarkosyl and r*Pf*MSP2 was purified by two rounds of nickel-chelate affinity chromatography. For r*Pf*MSP2 (FC27), an additional denaturation and glutathione-catalyzed refolding step^17^ was added to eliminate aggregation in the final product. We have successfully used this protocol to promote proper disulfide bond formation and restore conformational epitopes on several recombinant malarial proteins expressed in *E. coli*. Protein concentration was determined by bicinchoninic acid (BCA) protein assay (Pierce^TM^, Thermo Fisher Scientific). Purity and conformation were assessed under reducing and non-reducing conditions by SDS-PAGE and immunoblot analysis using anti-*Pf*MSP2 antibody (MRA-318, 1:5,000 dilution) or anti-*Pf*MSP8 IgG (1 μg/ml) as described above. The specificity of the rabbit anti-*Pf*MSP8 IgG has been established previously^15^.

### Assessment of fibril formation

(I). **Size exclusion chromatography**. *Pf*MSP2 and *Pf*MSP2/8 recombinant proteins were evaluated for presence of monomeric and oligomeric populations via size exclusion chromatography on a Superdex® 75 matrix (GE Health Care Life Sciences, Marlborough, MA). The column was equilibrated in TBS and calibrated using ribonuclease A, ovalbumin and conalbumin standards (GE Health Care Life Sciences). Immediately after thawing, 1 mg of purified recombinant protein was applied to the column at a flow rate of 0.5 ml/min and absorbance of the eluate at 214 nm monitored.
(II). **Thioflavin T fluorescence assay**. To assess amyloid-like fibril formation, recombinant proteins (10 μM, in PBS) were co-incubated with 30 μM Thioflavin T (ThT; Thomas Scientific, Swedesboro, NJ) in the presence or absence of 40 μM epigallocatechin-3-O-gallate (EGCG; Millipore Sigma), an inhibitor of fibril formation^26,29,48^. Samples were transferred in duplicate to a 96-well black microwell plate (Thermo Fisher Scientific, 300 μl /well) and incubated at 37 °C for 98 hours. PBS alone and PBS with 30 μM ThT served as negative controls. Fluorescence was measured (443 nm excitation, 484 nm emission) at 0, 2, 4, 20, 44 and 98 hours of incubation on a Hitachi F-7000 Fluorescence Spectrophotometer. Duplicate samples were averaged and blanked against PBS with Thioflavin T.

### Mouse splenocyte proliferation assay and TLR2 neutralization

Male CB6F1/J (BALB/cJ X C57BL/6 J) mice were purchased from The Jackson Laboratory and housed in the Animal Care Facility of Drexel University College of Medicine. Animal studies were reviewed, approved and conducted in compliance with Drexel University’s Institutional Animal Care and Use Committee (Protocol No. 20645) and in accordance with the US Public Health Service (PHS) Policy on Humane Care and Use of Laboratory Animals. Naïve splenocytes were prepared as described previously and stimulated in triplicate with 10 μg/ml of recombinant antigen, 1 μg/ml of concanavalin A (ConA, Millipore Sigma) or left unstimulated^15,16,61^. Proliferation was measured in the presence of anti-TLR2 blocking monoclonal antibody or an IgG1 isotype control (1 μg/ml; InvivoGen, San Diego, CA). After 78 hours of incubation at 37 °C in 5% CO_2_, 1 μCi of [^3^H]thymidine (PerkinElmer Life and Analytican Sciences; Shelton, CT) was added to each well, and cells were harvested after an additional 18 hours. Based on [^3^H]thymidine incorporation, a stimulation index for each condition was calculated as the mean counts per minute of stimulated samples divided by the mean counts per minute of unstimulated samples.

### Generation of polyclonal rabbit antisera

Polyclonal rabbit antisera were generated by Lampire Biological Laboratories (Pipersville, PA). Adult New Zealand White rabbits (n = 4) were immunized subcutaneously with 100 μg of recombinant *Pf*MSP2 or *Pf*MSP2/8 emulsified in Montanide ISA 720 (0.5 ml; Seppic Inc., Paris, France) at a ratio of 70:30 (vol/vol). One control rabbit received adjuvant alone. Two booster immunizations were administered at 4-week intervals. Test bleeds were taken two weeks after primary and secondary immunizations and terminal sera were collected four weeks after the tertiary immunization. The specificity of newly generated rabbit anti-*Pf*MSP2 and anti-*Pf*MSP2/8 is established in Figs 4–7.

### *P. falciparum in vitro* culture and assays of native *Pf*MSP2

*Plasmodium falciparum* blood-stage parasites (NF54 and D10 strains) were maintained as previously described^17^.

(I). **Immunoblot of parasite lysate**. A mixed blood-stage lysate from asynchronous *P. falciparum* infected RBCs^15,17^ (2.3 × 10^7^ parasite equivalents per lane) was separated by SDS-PAGE on 12% gels under reduced and non-reduced conditions, blotted and probed with rabbit anti-r*Pf*MSP2/8 and anti-*Pf*MSP2 sera diluted 1:2500. Adjuvant control rabbit sera served as a negative control. Bound antibodies were detected as above.
(II). **Indirect immunofluorescence assay**. Thin blood smears of *P. falciparum* NF54-infected RBCs were prepared and fixed in acetone-methanol (1:1)^15,62^. Slides were incubated with pooled r*Pf*MSP2/8 or r*Pf*MSP2 antisera or adjuvant control sera (1:400) for 30 minutes at 37 °C. Bound antibody was detected by fluorescein isothiocyanate (FITC)-conjugated goat anti-rabbit IgG (5 μg/ml; Invitrogen). Slides were mounted with SlowFade Gold antifade reagent with DAPI (4′,6-diamidino-2-phenylindole; Life Technologies, Thermo Fisher Scientific). Images were acquired using an Olympus BX60 fluorescence microscope (Olympus America, Inc.; Melville, NY) and SensiCam QE cooled digital 12-bit charge-coupled-device (CCD) camera system (PCO-Tech, Inc.; Romulus, MI), and analyzed using SlideBook 5.0 software (Intelligent Imaging Innovations, Inc.; Denver, CO).

### Quantification of antigen-specific antibody by ELISA

(I). **Direct binding ELISA**. Antigen-specific antibody titers generated by *Pf*MSP2-based vaccines were quantified by direct-binding ELISA^17^. ELISA plates were coated with 0.25 μg/well of r*Pf*MSP2 (3D7), r*Pf*MSP2 (FC27), r*Pf*MSP2/8 or r*Pf*MSP8 and incubated with 2-fold serial dilutions of individual rabbit sera. High-titer pooled sera included on each plate were used for normalization. Bound antibody was detected by HRP-conjugated goat anti-rabbit IgG with ABTS [2,2′-azino-bis(ethylbenzothiazoline-6-sulfonic acid) diammonium salt] as substrate. A_405_ values between 1.0 and 0.1 were plotted, and titer was calculated as reciprocal of the dilution that yielded an A_405_ of 0.5. For peptide ELISAs, four representative peptides (18-mers) containing characterized B cell epitopes (underlined) were synthesized (RP-HPLC purity > 90%; GenScript, Piscataway, NJ). The four selected peptide sequences were as follows: N-terminal conserved region peptide (FINNAYNMSIRRSMAESK), central variable region (CVR) peptide A (TNPKGKGEVQEPNQANKE), CVR peptide B (DSQTKSNVPPTQDADTKS) and C-terminal conserved region peptide (ECTDGNKENCGAATSLLN). Plates were coated with 0.5 μg/well of each peptide and incubated with individual rabbit sera (1:200 dilution). Bound antibody was detected as above.
(II). **Competition ELISA**^15^. Each rabbit serum was diluted to yield an A_405_ of ~1.0. Anti-r*Pf*MSP2 (3D7) or anti-*Pf*MSP2/8 sera were pre-incubated with 10-fold increasing concentrations (1 pM-1 μM) of r*Pf*MSP2 (3D7) or r*Pf*MSP2/8 in buffer containing 20 mM Tris-HCl (pH 8.0), 50 mM NaCl, 5 mM EDTA, 0.5% Triton-X-100, and 0.5% deoxycholate at 4 °C, overnight. All antibody-antigen mixtures also contained 1 μM of r*Pf*MSP8 to completely adsorb anti-r*Pf*MSP8-specific antibodies. The antibody-antigen samples were then added to ELISA wells coated with r*Pf*MSP2/8 or r*Pf*MSP2 (3D7) and binding of non-complexed, free antibodies was quantified as described above. Percent inhibition was calculated as [1 − (A_405_ of IgG in the presence of inhibitor/A_405_ of IgG in the absence of inhibitor)] × 100.

### *P. falciparum* growth inhibition assay

A 1.5 ml sample of each rabbit serum was heat inactivated and adsorbed against human O + RBCs prior to purification of the IgG fraction by Protein G affinity chromatography^63^. The ability of purified rabbit anti-*Pf*MSP2 and anti-*Pf*MSP2/8 IgG (5.0 mg/ml and 2.5 mg/ml) to inhibit the *in vitro* growth of *P. falciparum* (NF54) was measured in a standard growth inhibitory assay (GIA)^64^. Rabbit anti-*Pf*MSP1/8 and adjuvant control IgG were used as positive and negative controls, respectively. Parasite growth inhibition was based on measurement of parasite lactate dehydrogenase activity relative to *P. falciparum* incubated with RBCs in the absence of rabbit IgG.

### Opsonophagocytosis Assay

(I). **THP-1** ***in vitro*** **culture**. THP-1 cells (ATCC^®^ TIB-202) were purchased and maintained in RPMI 1640 (ATCC 30-2001) supplemented with 0.05 mM 2-mercaptoethanol and 10% fetal bovine serum. Cell density was regularly monitored and maintained between 1 × 10^5^ and 1 × 10^6^ cells/ml. Cells were passaged when cell density reached 8 × 10^5^ cells/ml and were not used beyond passage ten.
(II). **Merozoite isolation and preparation**. For isolation of merozoites, *P. falciparum* cultures were synchronized by treatment with 5% D-sorbitol. Late stage, pigmented trophozoites were isolated by magnetic column purification^65^ and subsequently cultured with complete culture medium supplemented with 10 μM trans-epoxysuccinyl-L-leucylamido-(4-guanidino) butane (E64, Millipore Sigma) for 6–8 hours. Merozoites were mechanically released from late stage, fully segmented schizonts^15^ and free hemozoin removed by a second passaged over a magnetic column. Purified merozoites were pelleted and stained with 5 μM Nuclear Red^TM^ LCS1 (AAT Bioquest, Sunnyvale, CA) for 15 minutes at 4 °C. Stained merozoites were washed and fixed with 2% formaldehyde (Polysciences Inc., Warrington, PA). Fixed, stained merozoites were washed and counted using CountBright^TM^ Absolute Counting Beads (Thermo Fisher Scientific) on a BD Accuri^TM^ C6 (BD Biosciences) flow cytometer. Merozoites were resuspended at 1 × 10^7^ merozoites/ml in THP-1 culture media and used as described.
(III). **Opsonization and Phagocytosis**. U-bottom, 96-well plates were pre-coated with FBS (1 hr at room temperature). For opsonization, 30 μl merozoites (1 × 10^7^ merozoites/ml) were transferred to each well and incubated with 3 μl of test IgG at a concentration of 0.1 mg/ml or 0.25 mg/ml for 1 hr at room temperature. Unopsonized and control IgG opsonized merozoites served as negative controls. Opsonized merozoites were washed, resuspended in 150 μl THP-1 medium and plated in triplicate (50 μl/well). THP-1 cells were resuspended at 2 × 10^5^ cells/ml, and 100 μl were co-incubated with opsonized merozoites for 10 minutes at 37 °C. Phagocytosis was stopped by the addition of 50 μl cold PBS containing 3% FBS. THP-1 cells were washed, fixed in 2% formaldehyde and analyzed on BD Accuri^TM^ C6 flow cytometer using FlowJo® analysis software. The level of phagocytosis was determined as percentage of THP-1 cells positive for Nuclear Red fluorescence relative to unopsonized negative control. Percent phagocytosis of adjuvant control IgG was subtracted from concentration-matched test samples.

### Statistical analysis

In the comparison of two, non-paired samples, Mann-Whitney test was used to determine statistical significance of differences between antigen-specific IgG titers. To determine statistical significance of boosting in antigen-specific IgG titers, the Friedman test followed by Dunn’s post hoc test was used. Considering sample sizes, nonparametric tests were used. Differences were considered significant with probability (*p*) values ≤ 0.05.

### Equipment and Settings

(I). **Coomassie-stained polyacrylamide gels**. Coomassie Blue-stained polyacrylamide gels were imaged using the ImageQuant^TM^ LAS 4000, gel documentation camera (GE Healthcare Life Sciences Technology, Marlborough, MA). Images were imported into Microsoft PowerPoint for mock up. Brightness and contrast were adjusted to increase clarity of unstained portion of gel; adjustments were applied to entire gel equally. Full-length gels are shown in Figs 1B,D, 2A,D.
(II). **Immunoblots**. Chemiluminescent immunoblot images were acquired by exposure to HyBlot CL® Autoradiography Film (Denville Scientific Inc., Metuchen, NJ). Developed films were scanned using an HP Scanjet G4050 scanner, and scanned images were imported into Microsoft PowerPoint for mock up. Minimal adjustments to brightness and contrast were made increase clarity of the gray film background. All adjustments were applied to the entire gel equally. Full-length gels shown in Figs 1C,E, 2B,C,E and 5A.
(III). **Microscopy**. Images were acquired at a resolution of 1376 × 1040 pixels using an Olympus BX60 fluorescence microscope with a UPlanApo (100×/1.35) oil immersion objective lens (Olympus America, Inc.; Melville, NY) and SensiCam QE cooled digital 12-bit charge-coupled-device (CCD) camera system (PCO-Tech, Inc.; Romulus, MI), and analyzed using SlideBook 5.0 software (Intelligent Imaging Innovations, Inc.; Denver, CO). Representative images (Fig. 5B) were imported into Microsoft PowerPoint for mock up. Edges were cropped to the focal point of the image to eliminate blank space.

For all gel, immunoblot and microscopy images, Microsoft PowerPoint figures were saved in ‘Press Quality’ PDF format and imported into Adobe Photoshop v5.5 at a resolution of 600 dpi. In Photoshop, the image was sized, flattened and saved in a Tagged Image File Format (TIFF) with Image Compression set to LZW, Pixel Order set to Interleaved and Byte Order set to IBM PC. No additional adjustments were made to any of the images in Photoshop.

## Data Availability

The data generated and analyzed that support the scientific findings and claims of this study are presented in this published article.

## Supplementary information

Supplemental Data
