# Supplementary material for: Immunization with merozoite surface protein 2 fused to a Plasmodium-specific carrier protein elicits strain-specific and strain-transcending, opsonizing antibody
Source: Sci Rep. 2019 Jun 21;9:9022. doi: 10.1038/s41598-019-45440-4 (PMC6588637; doi:10.1038/s41598-019-45440-4)
Supplement: Supplementary file 1 — Supplemental Data [file 41598_2019_45440_MOESM1_ESM.pdf]

Supplementary Material

Immunization with merozoite surface protein 2 fused to a *Plasmodium*-specific carrier protein elicits strain-specific and strain-transcending, opsonizing antibody

Jacqueline S. Eacret, Donna M. Gonzales, Raymond G. Franks and James M. Burns Jr.*

Center for Molecular Parasitology, Department of Microbiology and Immunology, Drexel University College of Medicine, 2900 Queen Lane, Philadelphia, PA 19129, USA

*Corresponding author:

Email: jmb53@drexel.edu

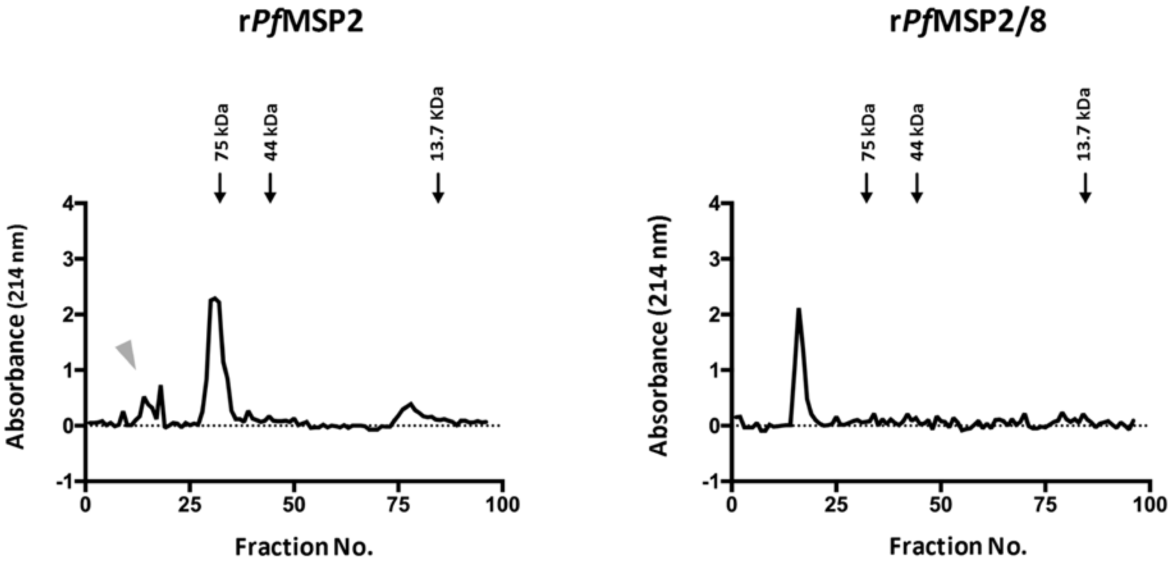

Figure S1. Assessment of fibrillar content for r*Pf*MSP2 and r*Pf*MSP2/8 purified antigens. Aliquots of purified r*Pf*MSP2 and r*Pf*MSP2/8 were thawed and immediately subjected to size exclusion chromatography to assess fibrillar content of stored antigens used for immunization of rabbits. Fractions were collected (0.5 ml) and absorbance was measured at 214 nm. Black arrows correspond to peaks generated by low molecular weight protein standards conalbumin (75 kDa), ovalbumin (44 kDa) and ribonuclease A (13.7 kDa). Grey arrow indicates protein aggregates.

Supplementary Table 1

Anti-*Pf*MSP8 IgG is not functional in the OPA

		% Phagocytosis	
**Sample**	**Rabbit antiserum**	**0.25 mg/ml IgG**	**0.1 mg/ml IgG**
**Test IgG**	*Pf*MSP8 IgG	2.5	1.7
**Assay (+) cont.**	*Pf*MSP2/8	32.8	

Concentration-matched adjuvant control subtracted as background
